# Prader–Willi Syndrome and Weight Gain Control: From Prevention to Surgery—A Narrative Review

**DOI:** 10.3390/children10030564

**Published:** 2023-03-16

**Authors:** Valeria Calcaterra, Vittoria Carlotta Magenes, Francesca Destro, Paola Baldassarre, Giustino Simone Silvestro, Chiara Tricella, Alessandro Visioli, Elvira Verduci, Gloria Pelizzo, Gianvincenzo Zuccotti

**Affiliations:** 1 Pediatrics and Adolescentology Unit, Department of Internal Medicine, University of Pavia, 27100 Pavia, Italy; 2 Pediatric Department, “Vittore Buzzi” Children’s Hospital, 20154 Milan, Italy; 3 Pediatric Surgery Department, “Vittore Buzzi” Children’s Hospital, 20154 Milan, Italy; 4 Department of Health Sciences, University of Milan, 20142 Milan, Italy; 5 Department of Biomedical and Clinical Science, University of Milan, 20157 Milan, Italy

**Keywords:** Prader–Willi syndrome, weight gain, diet, exercise, hormonal therapy, bariatric surgery

## Abstract

Severe obesity remains one of the most important symptoms of Prader–Willi Syndrome (PWS), and controlling weight represents a crucial point in the therapeutical approach to the syndrome. We present an overview of different progressive patterns of growth that involve controlling weight in PWS. Mechanisms involved in the development of obesity and in preventive and therapeutic strategies to control weight gain are discussed. Early diagnosis, a controlled diet regimen, regular physical activity, follow-up by multidisciplinary teams, and hormonal treatment improved the management of excessive weight gain. In selected cases, a surgical approach can be also considered. Controlling weight in PWS remains a challenge for pediatricians. The importance of consulting different healthcare specialists, starting from the neonatal and pediatric age, is also considered as a crucial approach to controlling weight, as well as to limiting and preventing the onset of obesity and its complications.

## 1. Introduction

Prader–Willi Syndrome (PWS) is a complex genetic disorder that occurs in about 1 in 15.000 subjects. PWS is caused by the absence of paternally expressed imprinted genes at 15q11.2-q13 through paternal region deletion (about 65–75% of individuals), maternal uniparental disomy 15 or both 15 s from the mother (about 35%), or an imprinting defect (1–3%) [1].

The multisystemic manifestations of PWS occur throughout the patient’s life, starting from the neonatal period. Affected neonates present with hypotonia and feeding disorders (poor sucking/failure to thrive) followed by excessive weight gain and compulsive hyperphagia that lead to progressive life-threatening obesity occurring early in childhood, unless properly addressed. Indeed, PWS is considered the most frequent genetic cause of severe eating disorders [1,2,3].

Obesity in PWS is usually resistant to dietary, lifestyle, and pharmacological treatment, and progressively determines severe cardiologic, endocrine, respiratory, metabolic and orthopedic complications and hyperphagia-related behaviors, with increased risks of gastrointestinal perforation, aspiration, choking and swallowing difficulties, and reduced gastrointestinal motility [1,4]. Morbidity and mortality (around 1.5% per year) are essentially linked to the consequences of severe obesity [1,4].

An early diagnosis allows for the timely management of patients and the activation of a specific and dedicated multidisciplinary care regimen that has been shown to favorably affect the natural evolution of the syndrome. Establishing a collaborative approach to management between doctors and families/caregivers is essential, demonstrating the need for a multidisciplinary therapeutic program [5]. Early diagnosis and a multidisciplinary approach to care have improved developmental outcomes thanks to early access to specific treatments aiming to help patients with PWS reach a better quality of life [4,6].

In this narrative review, we present an overview of different progressive patterns of growth that involved controlling weight in PWS. Mechanisms involved in the development of obesity and in the preventive and therapeutic strategies to control weight gain are discussed. The importance of consulting different healthcare specialists, starting from the neonatal and pediatric ages, was also considered as a crucial approach to controlling weight and limiting and preventing the onset of obesity and its complications.

## 2. Methods

We presented a literature review of a narrative nature [7]. Focusing on multidisciplinary treatment and management to control weight in PWS, reviews, original papers, metanalyses, and clinical trials published from 2000 onwards were reviewed. Case reports were excluded, as well as case series, descriptive studies, letters, commentaries, and articles that had no full-text accessible in English. We performed a non-systematic search in the PubMed, Scopus, and Web of Science databases. The following terms (alone and/or in combination) were used for the literature search: Prader-Willi, nutrition, weight, endocrine, endocrinological, therapy, physical activity, pharmacological treatment, and weight gain. The contributions were collected by V.C.M., F.D., P.B., G.S.S., A.S., and C.T.; they were then critically reviewed by V.C. and E.V. and discussed by V.C., E.V., G.P., and G.Z. All authors approved the final version.

## 3. Pattern of Growth in Children with PWS

**Figure 1:**
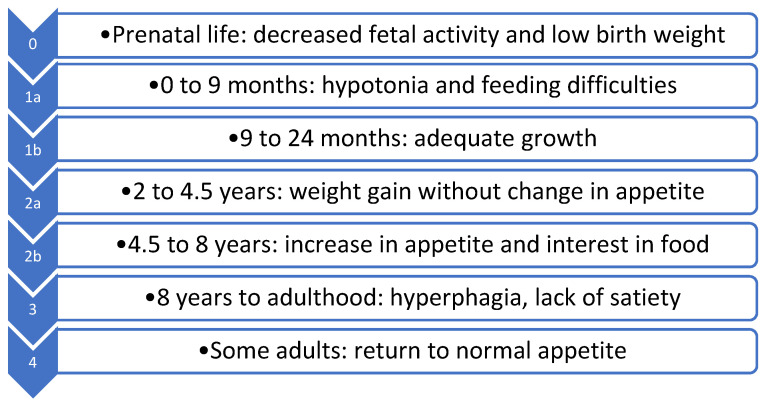
PWS nutritional phases [8].

PWS is characterized by different patterns of growth with gradual progression that involve the need for controlling weight [8] (Figure 1).

Phase 0 (prenatal life) is characterized by decreased fetal activity, polyhydramnios, breech presentation, small for gestational age, and lower birth weight than siblings [4,9].

Phase 1 is subdivided into the following phases: phase 1a (0 to 9 months), characterized by hypotonia at birth, poor sucking reflex, and feeding difficulties (infants in this phase do not show desire to eat, so failure to thrive may develop), and phase 1b (9 to 24 months), in which appetite returns to normal and infants grow steadily along a specific growth chart [4,9].

Phase 2 involves the transition from anorexia to excessive weight gain and is subdivided into phases 2a and 2b [4,9]. Phase 2a (2–4.5 years) is characterized by weight gain without a significant change in appetite. During this phase, appetite is appropriate for age, gastric motility tends to slow down, and metabolism begins to change [4,9]. This weight gain is hypothesized to be a result of decreased resting energy expenditure (REE) and possibly abnormalities in carbohydrate metabolism [10]. Phase 2b (4.5–8 years) is then characterized by an increase in appetite and interest in food [4,9].

Phase 3 (8 years–adulthood) involves hyperphagia with uncontrolled appetite, lack of satiety, and increased food-seeking behavior.

Finally, some adults move on to Phase 4, where their appetite is no longer insatiable [4,9].

Progressing through these phases, patients develop behavioral and endocrine dysfunctions.

Of these, severe obesity, arising from hypothalamic abnormalities of satiety and hormonal disorders, is the main problem in children with PWS, and it is often accompanied by complications, such as type 2 diabetes, dyslipidemia, obstructive sleep apnea syndrome, and right-side heart failure [4,9,11].

**Figure 2:**
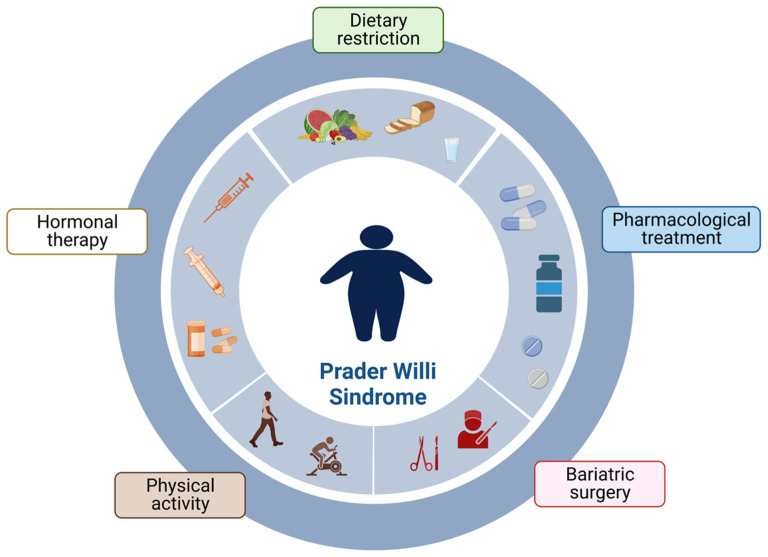
Approaches to weight gain control in PWS.

Endocrinologists and nutritionists, as part of an interdisciplinary team, play a crucial role in obesity management in PWS, considering that restricted diet and hormonal treatments are the most successful approaches to reducing and maintaining body weight [5,11]. In selected conditions, surgery could be also considered (Figure 2).

## 4. Hormonal Features and Therapeutical Approach

Hypothalamic dysfunction and multiple endocrine abnormalities are typical features of PWS [1].

The mechanism of obesity in PWS is not completely known and requires a complex interaction of a variety of factors. Certainly, hypothalamic disruptions to pathways of satiety control and aberrations in hormones regulating appetite/satiety, along with abnormal eating behaviors (compulsive food-seeking and food-stealing), result in a vicious circle of perpetuating obesity that represents a major complication of this syndrome.

Changes in certain brain areas play a significant role in this process. In particular, many functional magnetic resonance imaging studies showed a greater post-meal stimulation of food activation brain centers in the limbic and paralimbic region (amygdala, hippocampus, and hypothalamus) and a lower activation in cortical inhibitory circuits (medial prefrontal and orbitofrontal cortex) [12]. These alterations confirmed the dysregulation of reward pathways and the impairment in inhibitory cortical regions, resulting in hyperphagia and obesity development in PWS.

In the literature, many orexigenic and anorexigenic substances are investigated as being responsible for appetite disorders. Ghrelin is a hormone produced by gastric mucosa which normally simulates short-term food intake during starvation (orexigenic hormone); PWS results in elevated ghrelin levels and that are not appropriately suppressed after eating [4,13]. Beauloye et al. showed that hyperghrelinemia occurs before the onset of hyperphagia due to the presence of the inactive form of ghrelin (AUG) that has an opposite effect, explaining the anorexia typical of the first nutritional phases of the syndrome [14]. Later, persistently high serum levels of ghrelin play a central role in promoting hyperphagia, increasing appetite, weight gain, and obesity, and, thus, increasing the risk of obesity-related comorbidities, such as DMT2, dyslipidemia, and cardiovascular disease, especially in adult life. Obestatin is a peptide derived from the proteolytic cutting of the primary gene transcript encoding for the hormone ghrelin and produces the opposite effect [15]. Normally, it suppresses appetite and reduces weight. There are limited data on the role of obestatin in PWS, and no study has demonstrated the difference between PWS patients and obese controls [15]. Park et al. evaluated 15 PWS children and 18 controls during an oral glucose tolerance test in order to analyze the changes in the obestatin levels after glucose loading and to characterize the correlations between obestatin, ghrelin (total and acylated), and insulin [16]. The researchers measured the plasma levels of obestatin, ghrelin, and serum insulin at 0, 30, 60, 90, and 120 min after glucose challenge, and calculated the areas under the curves (AUCs) [16]. The results showed no significant difference in the AUC of the plasma obestatin between the PWS children and normal obese controls (*p* = 0.885), demonstrating that obestatin is not regulated by insulin both in PWS children and in obese controls [16]. Other hormones involved are pancreatic polypeptides (PP), which reduce the post-meal stimulation of activation centers in patients with PWS, and peptide YY (PYY), with debated effects [11,13].

Among the plasmatic adipocytokines involved in the control of appetite and adiposity, no differences in leptin values are present between PWS patients and obese and healthy normal weight controls. Adiponectin concentrations, however, are markedly higher in PWS patients and are associated with an increased insulin sensitivity, whereas elevated resistin values are related to lipogenesis and potentially to insulin-resistance in obese PWS patients [17,18].

PWS is characterized by hypothalamic impairment, which results in many of the typical manifestations, including hyperphagia, instable body temperature, high pain threshold, disordered breathing during sleep, and numerous endocrine findings [4,19,20]. The latter include growth hormone deficiencies (GHD), hypogonadism, hypothyroidism, central adrenal insufficiency, and complications of obesity, such as type 2 diabetes mellitus (T2D), which contribute to the abnormal body compositions and weight of PWS patients [4,19,20].

About 40–70% of children with PWS are affected by GHD, resulting from an inadequate response to GH stimulation testing, a decreased spontaneous 24 hour GH secretion, and low insulin-like growth factor 1 (IGF-1) values [4,21,22,23]. The most common clinical manifestations related to this condition include short stature, increased fat mass (in particular truncal fat) determining weight gain, poor muscle tone and strength, and REE and exercise tolerance.

In 2000, the Federal Drug Administration (FDA) approved recombinant human GH (rhGH) in the United States for the treatment of GHD in PWS, and, in 2001, it was approved by the European Medicine Agency (EMA) in Europe [24]. The ideal age to begin this therapy is not established; moreover, the initiation of therapy before the onset of obesity seems to be more effective [25,26]. In particular, guidelines suggest starting at 3 months and surely before 2 years with an initial dose of 0.5 mg/m^2^/die with subsequent adjustments until 1 mg/m^2^/die [25,26]. The beneficial effects of rhGH have improved many aspects of this syndrome which may persist into adult life. Initially, it was hypothesized that this treatment acted only by improving the final height and growth rate of these patients, but many studies have confirmed the role of GH as an anabolic agent. In particular, rhGH modifies the body’s composition, increasing lean mass and reducing fat mass with a subsequent decrease in body mass index (BMI) and improvements in fat expenditure with greater muscle mass [27]. Carrel et al. demonstrated that in 48 children with PWS, a number of 27 patients treated with GH for 6 years showed modification in body fat (36.1% vs. 44.6%) and lean body mass (24.1 kg vs. 16.7 kg) compared to untreated children [28]. Metabolic changes were also demonstrated in these patients, such as improvements in lipid profiles and non-significant increases in fasting glucose. Other effects included a marked improvement in motor development with a better exercise capacity and strengthening physical performance but also in cognitive development in areas, such as language ability and better relationship and socialization skills. Recently, Ayet-Roger et al. compared the cognitive and adaptive performance of 31 patients with genetically confirmed PWS patients grouped in two cohorts, one treated with GH before 2 years old (Group 1) and the other receiving the treatment later (Group 2) [29]. The researchers evidenced that patients treated earlier (Group 1, n = 10) obtained higher and statistically significant scores in Total Intelligence Quotient (TIQ), General Ability Index (GAI), and General Adaptive Behavior (GAB), implying better cognitive and adaptive performance compared to patients receiving GH later (Group 2) [29].

Generally, this replacement therapy is well tolerated, but it is not without risk. There are few data about its potential complications, such as respiratory problems and obstructive apnea and the development of scoliosis or worsening of a pre-existing form of it. Before starting treatment, there are some recommendations, such as an otolaryngology evaluation if there is a history of sleep disorders, snoring, or adenoid and tonsillar hypertrophy; a respiratory assessment; an analysis of body composition; and an accurate imaging of the spinal column with eventual orthopedic referral if marked scoliosis is present. The real contraindications to therapy include uncompensated diabetes, uncontrolled severe OSA, severe obesity, active psychosis, and malignance. Another aspect to remember is periodically monitoring clinical conditions of these patients and their serum levels of insulin-like growth factor-1 (IGF-1) every 6–12 months during therapy. Monitoring these serum levels is important because it has been widely described that high levels of IGF-1 are responsible for lymphoid hyperplasia, increasing the risk of OSA and a hypothetical risk of malignancy [21,24,30]. The combination between GH replacement and lifestyle interventions constitutes the main determinants to preventing obesity in childhood.

Hypogonadism represents another typical feature of this syndrome present in both sexes. It is characterized by multifactorial etiology as result of an association of primary gonadal and hypogonadotropic hypogonadism [31]. In hypogonadism, hormone replacement therapy (HRT) [31] represents the main treatment to stimulate puberty and improve the sexual function of PWS subjects.

PWS males have a normal mini-puberty during infancy with normally elevated luteinizing hormone (LH), follicle-stimulating hormone (FSH), and testosterone levels within the first few months of life [25,31,32,33]. After this mini-puberty, gonadotropins and testosterone decrease to pre-pubertal levels. Typically, males develop a delayed or incomplete puberty and consequently commonly become infertile in adulthood [25,31,32,33]. Cryptorchidism (either unilateral or bilateral) is almost universal in PWS males, ranging from 66% to 100% of newborns with PWS, and it requires orchiopexy to reposition the testicles in their correct anatomical location. However, many studies have demonstrated that an early treatment with human chorionic gonadotropin (hCG) may promote spontaneous testicular descent and also increase penile length and scrotal size [25,31,32,33]. In clinical practice, testosterone injections started at 15–16 ages at lower doses until doses were tolerated well and under close supervision of an endocrinologist [25,31,32,33,34,35]. Other formulations of testosterone exist which are more expensive and require a daily administration. PWS males undergoing HRT are infertile [36], and no cases of paternity have been detected.

About 76% of PWS females are born with hypoplasia of the external genitalia with labia minora and clitoral hypoplasia [34]. Puberty may occur at the normal age, but cases of delayed progression and impaired development have been described, and some girls manifest primary amenorrhea [2,20]. There is no standard care for the management of impaired development, and the effectiveness of supplemental gonadal hormones is still under debate due to the associated risk of developing or triggering behavior manifestations, such as aggressiveness, which may be already present in adolescents with PWS. Four cases of pregnancies have been reported in females with PWS, but the availability of published data is limited [24,25]. Moreover, risk of sexual abuse should be considered, as these patients are considered at high risk [37].

Studies indicate that inhibin B levels >20 pg/mL correlate with the potential for fertility in PWS females, though these levels are still frankly low [34,38,39].

In view of the wide spectrum of clinical manifestations present in women, substitution therapy should be as personalized as possible; therefore, it is important to know the patient’s residual hormonal activity before starting HRT. In particular, therapy with estrogen is indicated in cases of amenorrhea and oligomenorrhea, whereas combined treatment with progesterone and low levels of estradiol is recommended for women with amenorrhea [19,31].

The positive effects of sex steroids are widely described in the literature, for example, increasing bone mineral density and muscle mass and favoring an adequate development of secondary sexual characteristics [26,31,40]. No data on weight evolution during HRT have been reported. Further studies are necessary to better understand the exact underlying pathophysiology mechanism and to establish a unanimous standard management and therapy for these patients.

Concerning adrenal function, there is a high rate of premature adrenarche in both males and females with PWS, with a prevalence of 14–30%. Adrenarche may be associated with advanced bone age in some cases, although the presence of obesity may also cause skeletal advancement. Premature adrenarche in PWS is typically isolated and not rapidly progressive or associated with other signs of central puberty and, thus, represents a benign condition [20,41].

The prevalence of central adrenal insufficiency is not clear due to the different tests used across studies; therefore, there is not a standard consensus on how to best assess and manage this condition in PWS. In clinical practice, a dose of ACTH (Adrenocorticotropic hormone) and cortisol may be useful during an acute event, or patients may be submitted to stimulus tests to investigate a possible deficit in adrenal function. Several authors recommend a dose of prophylactic steroids only for significant surgical interventions, while others recommend it for all patients with PWS during a stressful event, including mild infections. It might also be useful in clinical practice to inform the family about the possibility of developing symptoms caused by adrenal insufficiency during stress and educate them on its management by giving adequate doses of hydrocortisone. However, it is important to remember that a chronic treatment could cause weight gain and increase the risk of hypertension and bone demineralization already present in PWS [4,20].

Another endocrine manifestation is central hypothyroidism, which may occur not only at birth but also during infancy. Prevalence of hypothyroidism is estimated at around 20–30% of patients, although some authors have reported this prevalence at only 2–4%. In addition to neonatal screening, it is important to investigate thyroid function after the first 3 months of life and then annually. During childhood, clinical manifestations given by thyroid deficiency range from reduced IQ, cognitive impairment, low mood, constipation, tiredness, drowsiness, weight gain, and reduced skeletal maturation [25,42]. The treatment of choice is replacement therapy with levotiroxina. PWS with untreated hypothyroidism showed negative effects in terms of the basal metabolic rate, BMI, and cardiovascular risk [35].

In patients treated with GH therapy, thyroid function should be monitored because studies have showed a marked reduction in fT4 with normal TSH levels and high or normal T3 values, assuming that GH therapy is responsible for a higher conversion activity of T4 to T3.

## 5. Nutritional Approach

### 5.1. Nutritional Management Early Childhood Phase

As reported in Figure 1, infants with PWS typically present with generalized hypotonia at birth [4,5,9]. Hypotonia usually affects the oral cavity as well, leading to a poor sucking ability and causing failure to thrive [1,43]. Breast milk should be offered anyway to these patients even though breastfeeding may not be optimal. In addition, these patients may benefit from the use of modified nipples to adjust the flow, and consultation with a breastfeeding expert may be useful [1,43]. Due to the poor sucking abilities of these children, the use of tube feeding is not unusual, but prolonged tube feeding and the absence of oral feeding might contribute to the already impaired speech ability seen in PWS patients [44].

Interestingly, Salehi et al. performed a retrospective review in order to investigate swallowing pathology in PWS infants by evaluating video-fluoroscopic swallow studies (VFSS) [45]. The authors studied 23 VFSSs performed on 10 PWS infants (average age 9.7 ± 8.4 months; range 3 weeks–29 months) and highlighted a high rate of swallowing dysfunction (pharyngeal residue 71%, aspiration events 87%). Moreover, all aspiration events detected were silent. These results, comparable to adult studies [46], indicate that swallowing dysfunction may be frequently present in PWS patients; thus, an evaluation of swallowing is essential in order to correctly address feeding difficulties [45]. Furthermore, this evaluation requires a multidisciplinary approach to optimize feeding safety in PWS [45].

In the first nutritional phases of PWS development, the goal is to promote growth, keeping the patient between the 50th and 75th weight percentiles and, thus, preventing malnutrition and avoiding becoming overweight [11,44]. Infants with PWS have a lower metabolic rate than healthy infants since they have lower lean mass and more fat mass, meaning that they require fewer calories [11,47]. This has to be taken into account by physicians and nutritionists [11,44].

The recommendations suggest that these infants should consume breast milk or formula milk until 6 months of age, and then purees may be slowly introduced, starting with a tablespoon and increasing over a few months [5]. As in typically developing children, a new food should be introduced on a weekly basis; then, around 10 months of age, formula may decrease to no more than 236.5 mL 3–4 times per day [5]. From phase 2a, children should start a balanced, low-calorie diet, bearing in mind that diet restrictions may lead to reduced intake of vitamins and minerals and, therefore, multivitamin supplements, especially vitamin D, may be important [5,11].

According to the scientific literature, the best nutritional strategy in these patients seems to be a well-balanced, low-calorie diet [11]. Different nutritional regimens have been proposed for these patients, but there are currently no specific recommendations suggesting the specific intake of carbohydrates, protein, or fiber for children with PWS [11]. In the future, methods to precisely measure energy expenditure, such as calorimetry, could be used to plan more accurate dietetic regimens [11].

Together with nutritional prescriptions, patients with PWS should be encouraged to perform regular physical activity in order to increase muscle mass (affected by hypotonia) and improve motor development [9]. Specifically, for children with PWS, the recommendations suggest 30 minutes of sustained activity three to five times per week [5].

### 5.2. Nutritional Strategies in Late Childhood/Adolescence

In early childhood, children affected by PWS rapidly gain weight, although no change in appetite is observed (indeed, appetite is considered appropriate for age). However, in the following phases, weight gain continues in parallel to an increase in appetite [8,48].

From about 8 years of age onward, children with PWS typically begin to suffer from hyperphagia, that is, the intense and unsatisfied desire to consume food, accompanied by unsatiety, food preoccupations and eating behavior problems [1,3,43]. Thus, individuals with PWS require control and supervision while eating, not only to prevent weight gain and associated early onset morbid obesity, but also to avoid the occurrence of choking, binges, stomach rupture, gastric necrosis, and—rarely—death [45,46,49].

Diet restrictions indeed become fundamental in these patients for the prevention of obesity, health complications, and early death [8,48]. In PWS patients, several nutritional regimens have been studied in order to limit caloric intake to less than recommended amounts for non-PWs children and adolescents of the same age and sex [8,48]. Indeed, when compared to age-, sex-, and BMI-matched controls, children with PWS show both a reduced REE and a reduced activity energy expenditure (AEE); this translates to a reduced caloric need (approximately 20% to 40% less calories) with respect to children without PWS to maintain fat mass [50,51]. Specifically, for preschool- and school-age subjects with PWS, the guidelines recommend from 10 to 11 cal/cm of length to maintain and 8 to 9 cal/cm to reduce body weight [11,44]. For young children, the recommendation is from 600 to 800 Kcal/day, while for adolescents and adults, it is from 800 to 1100 cal/day [11,47]. If subjects with PWS show obesity, it is necessary to frequently assess their body weight and to adjust their total caloric intake accordingly [11].

The best nutritional strategy in PWS seems to be a well-balanced, low-calorie diet [1,11,48]. In children with PWS, indirect calorimetry is not a routinely recommended method to measure REE, but it may be extremely useful to more precisely assess nutritional needs and plan a personalized nutritional intervention [1,11,48].

Interestingly, Miller et al. evaluated the effect of a calorie-restricted diet with modified macronutrient intake on weight and body composition in 63 children (aged 2–10 years) affected by PWS [1]. Two nutritional regimens were studied: one with a modified macronutrient distribution (45% carbohydrates, 30% fat, 25% protein, and at least 20 g of fiber per day), while the second one was only calorie-restricted (50% to 70% carbohydrates, 10% to 23% fat, 15% to 20% protein, and 12 g or less of fiber per day) [1]. The first regimen was followed by 33 children; the second one by 30 children [1]. Subjects consuming the first regimen showed significant improvements in both body fat (*p* < 0.0001) and weight management (*p* < 0.001) with respect to those on the calorie-restricted one [1]. Interestingly, in a recent work by Irizarry et al., the impact of carbohydrate restriction on hyperphagia, hormonal and metabolic balance, and adiposity in children with PWS was examined [52]; subjects consuming the low-carbohydrate diet were shown to have lower postprandial insulin levels, higher levels of fasting glucagon-like peptide 1 (GLP-1), increased gastric inhibitory peptide (GIP) concentrations, higher levels of postprandial GLP-1, and decreased ratio of fasting ghrelin to GLP-1 with respect to subjects on the low-fat diet [52]. In order to confirm these interesting results, further studies on larger samples are needed, but these first pieces of evidence suggest that a low-carbohydrate diet might limit food intake and improve glycemic control in PWS patients, causing an increase in GLP-1 and a reduction in the ghrelin-to-GLP-1 ratio [11]. Moreover, different works have shown that nondigestible carbohydrates, such as dietary fibers, through their action on the gut microbiome, may help PWS subjects [53,54]. The gut microbiota has been highlighted to be an important factor in obesity correlated to nutritional intake; thus, Zhang et al. conducted a hospitalized intervention trial in 38 obese children affected by PWS (n = 17) or simple, non-syndromic obesity (n = 21). The authors showed that obese children following a diet rich in non-digestible carbohydrates experienced a significant weight loss, together with structural changes in the gut characterized by increased acetate production from carbohydrate fermentation [54].

Furthermore, PWS subjects showed amelioration in behaviors correlated to hyperphagia (evaluated via the Dykens Hyperphagia Questionnaire) [54]. The reasons why such amelioration in behavior occurred have not been cleared up yet, but dietary fiber fermentation leads to an increase in short-chain fatty acids, which have been shown to raise levels of anorexigenic hormones, such as GLP-1 and pancreatic peptide YY (PYY) [54,55].

Overall, the studies cited demonstrate that children with PWS benefit from a well-balanced diet characterized by caloric restriction, high fiber intake, and low carbohydrate load [1,8,11]. Additional studies are needed in order to determine whether the action of dietary fibers on microbiota may be a potential treatment strategy to address hyperphagia and consequent overweight in children with PWS [48].

In addition, it is important to underline that subjects with PWS may need to take multivitamin supplements, and above all a vitamin D supplement, in order to compensate for the reduced intake of vitamins and minerals that may be suboptimal due to their restrictive diet [44,56,57]. Nowadays, there are no specific recommendations for a routine evaluation of vitamin D levels in these children, but an accurate assessment of vitamin D status and a tailored supplementation may be beneficial in these patients.

In conclusion, it is worth highlighting the theme of psychological food security in PWS. Indeed, this issue is an essential component in the management of obese children [58,59].

A description of this concept and a practical approach in PWS children has been extremely well addressed by Butler, Lee, and Whitman in the book Management of PWS [58]. Essentially, according to the authors, food security is achieved when food access is controlled to the extent that parents and children know with certainty which type of food they have (in terms of quality), how much (quantity), and when (timing) the patient will receive it [58].

Indeed, when food access is controlled following a precise nutritional plan, PWS children and their caregivers have no doubt about meals and snacks [58]. This leads to a proper weight management and a decrease in the psychological disturbances related to food insecurity [58,59].

## 6. Pharmacological Treatment

Pharmacologic therapy against hyperphagia and obesity in PWS is still under investigation, and currently no drugs tested have shown a significant efficacy in controlling appetite. There are many action mechanisms on the basis of obesity development in PWS, and potential drugs may act on all of them. Metformin, in addition to its insulin-sensitizing effect on the liver and muscle, acts centrally on the hypothalamic–pituitary pathway and also determines an increased intestinal production of GLP-1 [60]. A drug useful to reduce fat intestinal absorption is orlistat, a pancreatic lipase inhibitor indicated for obesity treatment, but few data about tolerance and efficacy are present for obesity in PWS. Other drugs evaluated are naltrexone and bupropion, which may synergistically stimulate hypothalamic neurons in hunger suppression, improving weight control. Some clinical trials have examined the use of GLP-1 agonists, such as liraglutide and exenatide, which cause glucose-dependent insulin pancreatic secretion, with the aim to inhibit appetite and weight gain. Many authors confirm their efficacy in the treatment of diabetic PWS subjects, improving BMI, waist circumference, and HbA1c values [20,61]. Moreover, GLP-1 agonists may aggravate delayed gastric emptying in persons with PWS [57]; thus, considering the reports of binge eating-induced and idiopathic gastric necrosis and fatal rupture in PWS [62], some caution in the use of these drugs and monitoring of gastric emptying during therapy should be considered [57]. Other drugs tested, such as sibutramine (a nonspecific inhibitor of serotonin and norepinephrine reuptake) and rimonabant (endocannabinoid 1 receptor antagonist) are instead retiring due to their associated adverse effects. Topiramate is an anti-convulsant therapy that has demonstrated improved behavioral aspects in PWS patients but with no effect on weight control [11].

To date, there are many drugs under investigation which impact on the lipid metabolism and the regulation of satiety/appetite pathways, such as intranasal oxytocin and oxytocin analogs, diazoxide, and beloranib [58,59]. Unfortunately, however, a randomized study on Beloranib was stopped due to venous thromboembolic events [63,64].

Livoletide, an unacylated ghrelin analog, was initially proposed to ameliorate hyperphagia in PWS [4,65]; however, the trials were discontinued due to lack of efficacy [64].

New potential anti-obesity strategies, already used in patients with hypothalamic obesity, such as centrally acting sympathomimetics [66], could be also considered in PWS [58].

## 7. Physical Activity

Children with PWS tend to have a sedentary attitude, with difficulty involving themselves in physical activities (PA) [67,68]. The effects of this lifestyle are reflected in both the cardiovascular fitness and muscular tone (already impaired for the typical body composition with higher adiposity and lower muscle mass and strength), inducing further reduction in their daily activities [67,68,69].

The promotion of physical activity is a well-known intervention strategy for obesity patients, but it has been associated with different results because the individual response (e.g., weight loss, body composition modifications) is widely variable [69].

Physical exercise has been prescribed as part of PWS obesity protocols as well, but the results remain unclear. Exercise training could improve physical fitness, but randomized controlled studies are needed to determine the type, length, and intensity of PA and its effective benefits [69].

In children and adolescents, acute response to exercise in PWS patients shows high heart rate (HR), slow HR recovery after submaximal exercise, and slow exercise efficiency [70,71,72,73]. Otherwise, HR after maximal exercise and blood pressure (BP) are similar to obese patients without PWS [71,74]. Coordination, agility, speed, muscular strength, and balance in PWS are impaired in comparison to non-PWS obese children [75]. Findings on hormonal changes during PA are non-uniform: catecholamine levels may or may not increase, and GH, lactate, free fatty acid, and glycerol levels remain similar after endurance PA, but glycerol and ketone seem to increase after lower body exercises [75,76,77].

The long-term effects of exercise in PWS seem promising in terms of improved cardiorespiratory response and performances after guided aerobic and resistance activities carried out for about 6 months with a frequency of 1 to 7 days per week [78,79]. Many authors have shown an improvement in the basal heart rate and recovery times [69].

The effects are less evident when PA is performed only at home [80,81], although one study reported improvements in terms of reduced inflammatory markers after a 24-week-long home-based program [82].

When compared to the general population, it is more difficult for PWS patients to achieve profound and long-lasting lifestyle changes.

It is probably for these reasons that body weight and composition changes occur in a very wide range (reported variations range from 0 to 2–12%) [69,80,82,83]. The presence of a well-structured nutritional plan (including length, composition, and adherence) should also be considered to analyze results [69,82].

Kaufman et al. showed improved endocrine profiles (reduced glucose and glycohemoglobin levels and better lipid metabolism) in PWS adults with type II diabetes after PA and nutritional interventions.

Evidence is also not uniform as far as posture, static balance, and coordination are concerned [83,84]. Specific training programs should be developed in order to perform more accurate analysis.

## 8. Surgical Treatment

Bariatric surgery (BS) is accepted as a therapeutic option in selected pediatric patients. Laparoscopic sleeve gastrectomy (LSG) was described as an effective and safe approach in this age group [85,86].

Recent data suggest that LSG has fewer nutritional risks than Roux-en-Y gastric bypass (RYGB) and avoids the adjustable gastric banding (AGB) foreign body and its potential associated complications [87,88].

However, ethical issues for pediatric and adolescent BS are currently being discussed in the international literature, especially concerning syndromic obesity, including PWS.

Reported experiences of bariatric surgery in PWS are limited, and there is no homogeneity regarding the different bariatric procedures.

Long-term outcomes and effects of BS for patients with PWS remain controversial because of the complex clinical picture, with various degrees of intellectual disability interfering with the patients’ compliance. Data regarding the effectiveness and long-term sustainability of weight loss and the risk/benefit ratio are still uncertain.

In 2015, Alqathani et al. analyzed the data of 24 PWS patients who underwent LSG, and reported effective weight loss and reduction in comorbidities [89]. Other BS procedures (e.g., gastric banding or bypass) have not shown encouraging results in terms of hyperphagia reduction or long-term weight loss and are associated with morbidity and mortality [90]. It is probably for this reason that previously reported series demonstrated poor weight loss efficacy and many more complications than those reported by Alqathani [91]. Indeed, LSG determines metabolic and endocrine modulation, influencing serum levels of GLP-1 and PYY (peptide YY) and ghrelin changes in terms of inhibition. On the other hand, malabsorptive procedures lack safety data and may lead to long-term complications.

On the contrary, for other types of hypothalamic obesity (e.g., craniopharyngioma), the efficacy of surgery may be reduced during long-term follow-up, more so after LSG rather than a modern Roux-en-Y bypass [92].

A recent review article on bariatric procedure outcomes among patients with PWS and other hyperphagic disorders showed the greatest weight loss within 1 year of surgery. The long-term outcomes reported are variable, and the authors suggest fewer durable results in comparison with other obesity patients [93]. For instance, Liu et al. noticed unsustainable long-term weight loss and no comorbidity resolution in PWS [94].

The role of home care and high-intensity constant support plays a crucial role in the long-term weight loss effect of BS [95].

Di Pietro et al. tried to address the issues of BS in PWS by analyzing the principles of healthcare ethics. The authors suggest being extremely cautious in indicating surgery, even in life-threatening conditions, and to fully assess the patient considering psychological, social, and ethical aspects [96]. There is increasing evidence supporting the need for pre-operative multidisciplinary evaluations and indicating the importance of close and long-term monitoring after surgery. Intellectual deficiencies and patients’ decision-making capacities are two of the most discussed ethical aspects [96]. Indeed, selection criteria for BS usually require physiologic maturity for decision making and active/voluntary involvement in the dietary and physical activity changes that are required to improve the outcomes [97,98].

An important concern regarding surgery in PWS patients is related to their anesthesiologic risk, characterized by an exaggerated response to hypnotic drugs, difficulties in ventilation due to facial dysmorphism, hypoxia problems, breathing control, and thermoregulation.

The ESPGHAN guidelines (Table 1) do not exclude patients with intellectual deficiencies from BS, suggesting the inclusion of an ethicist in the multidisciplinary team in order to evaluate each case individually. However, these guidelines also indicate the need for decisional capacity and the presence of a disability that would impair adherence to post-operative treatment as a surgical requirement and surgical contraindication, respectively [99]. The same indications are quoted by the Italian consensus position statement for the diagnosis, treatment, and prevention of pediatric obesity [100]. Similarly, there is no specification for surgery in PWS, but high anesthesiologic risk is considered a contraindication to performing bariatric operations.

According to the 2018 Guidelines of the American Society of Metabolic and Bariatric Surgery (ASMBS), developmental delay represents a surgical contraindication, but BS in PWS should be considered, especially when comorbidities exist [101]. These guidelines are based on the fact that mentally delayed individuals with clinically severe obesity and comorbidities are probably destined to fail in other weight management strategies.

In 2022, the ASMBS and the International Federation for the Surgery of Obesity and Metabolic Disorders (IFSO) published a revised list of indications for metabolic and BS [102] (Table 1). New knowledge about the disease and its consequences and surgical improvements have led to an expansion of surgical indications. Moreover, syndromic obesity, developmental delay, or autism spectrum diagnosis are no longer defined as surgical contraindications in adolescents. In all circumstances, a multidisciplinary team for perioperative evaluation and a long-term follow-up should be considered to reduce risks and improve outcomes.

The “Interdisciplinary European Guidelines on Metabolic and Bariatric Surgery” specifically refers to genetic syndrome and PWS patients as possible surgical candidates after multidisciplinary team evaluation [103].

Di Pietro et al. suggest considering BS in PWS as the last therapeutic option in case of life-threatening conditions, BMI ≥ 35 kg/m^2^ and severe comorbidities, or BMI ≥40 kg/m^2^ with more minor comorbidities [77]. Other parameters that should be evaluated are the effects of non-surgical weight loss options and the risk/benefit ratio. The management of patients requires pre-operative counselling and an adequate postoperative care system managed by a multidisciplinary group.

## 9. Conclusions

Severe obesity remains one of the most important symptoms of PWS, and controlling weight represents a crucial point in the therapeutical approach to the syndrome. Early diagnosis, a controlled diet regimen, regular exercise, follow-up by multidisciplinary teams, and hormonal treatment have improved the management of excessive weight gain. In selected cases, a surgical approach could be also considered. Controlling weight in PWS is a challenge for pediatricians. Multidisciplinary approaches and lifelong follow-up are the keys to ameliorating the quality of life of these patients and reducing the risk of obesity-related complications.

## Figures and Tables

**Table 1:** Guidelines for bariatric surgery.

Indications	SOCIETY (Publication Year)			
	ESPGHAN (2015)	ASMBS and IFSO (2022)	IFSO-EC and EASO (2013)	SIEDP and SIP (2018)
Bariatric surgery indications	BMI > 40 kg/m^2^ with severe comorbidities Type 2 diabetes mellitus Moderate-to-severe sleep apnea Pseudotumor cerebri NASH with advanced fibrosis (ISHAK score > 1) BMI > 50 kg/m^2^ with mild comorbidities Hypertension Dyslipidemia Mild obstructive sleep apnea Chronic venous insufficiency Panniculitis Urinary incontinence Impairment in activities of daily living NASH Gastroesophageal reflux disease Severe psychological distress Arthropathies related to weight	Recommend surgery: BMI ≥ 35 kg/m^2^, regardless of presence, absence, or severity of comorbidities. BMI ≥ 30 kg/m^2^ with T2D. Consider surgery: BMI of 30–34.9 kg/m^2^ after failure of nonsurgical methods. BMI thresholds in the Asian population: BMI ≥ 25 kg/m^2^.	18 to 60 years: 1.With BMI ≥ 40 kg/m^2^. 2.With BMI 35–40 kg/m^2^ and the following co-morbidities: Metabolic disorders. Cardiorespiratory disease. Severe joint disease. Obesity-related severe psychological problems. 3.BMI = current BMI or previously maximum attained BMI. 4.Reduce the BMI threshold by 2.5 for Asian individuals. 5.Patients with BMI ≥ 30 and <35 kg/m^2^ with T2D on individual basis. Children and adolescents: BMI > 40 kg/m^2^ (or 99.5th percentile) and at least one co-morbidity.	BMI ≥ 35 kg/m^2^ with at least one severe comorbidity: T2D. Moderate to severe obstructive sleep apnea (AHI > 15). Idiopathic endocranial hypertension. NAFLD with significant fibrosis (Ishak score > 1). BMI ≥ 40 kg/m^2^ with less serious comorbidities: Mild sleep apnea (apnea/hypopnea index > 5). Hypertension. Dyslipidemia. Carbohydrate intolerance.
Additional indications	Have attained 95% of adult stature. Have failed to attain a healthy weight with previously organized behavioral/medical treatments. Demonstrate commitment to psychological evaluation perioperatively. Avoid pregnancy for 1 year after surgery. Will adhere to nutritional guidelines after surgery. Have decisional capacity and will provide informed assent/consent, as age appropriate.	No upper patient age limit after careful assessment of co-morbidities and frailty. Individuals considered higher risk for general surgery may benefit from MBS Evaluation by a multidisciplinary team Developmental delay is not a contraindication Children and adolescents: BMI > 120% of the 95th percentile with a major co-morbidity BMI > 140% of the 95th percentile, after multidisciplinary team evaluation	Weight loss before surgery is not a contraindication. Regain weight after conservative management is a surgical indication. Children and adolescents: At least 6 months of weight reducing attempts in a specialized center. Skeletal and developmental maturity. Commitment to comprehensive medical and psychological evaluation. Participation in a post-operative multidisciplinary program. Access to a unit with specialist pediatric support. CONTRAINDICATIONS: 1. Absence of medical management. 2. Impossible prolonged medical follow-up. 3. Non-stabilized psychiatric and eating disorders. 4. Alcohol and/or drug abuse. 5. Diseases threatening life in the short term. 6. Subjects unable to care for themselves. 7. Secondary diabetes. 8. Antibodies positive or C-peptide < 1 ng/ml or unresponsive to mixed meal challenge.	Skeletal (95% of adult stature) and sexual maturation. Long lasting severe obesity. Previous failure of any conservative intervention (at least 12 months). Family and social support. Decisional capacity for surgical management and the post-surgery follow-up. Able to express informed assent. Surgery should be performed in a highly specialized center with a multidisciplinary team. CONTRAINDICATIONS: 1. Substance abuse (drugs/alcohol). 2. Incapability of self-care. 3. Low compliance to long-term follow-up. 4. Reduced life expectancy. 5. High anesthesiologic risk. 6. Inflammatory bowel disease. 7. Pregnancy (including pregnancy 2 years after surgery).
Specific considerations on PWS	N/A	N/A	Bariatric surgery can be considered in genetic syndromes, such as PWS, only after careful consideration of an expert medical, pediatric, and surgical team.	PWS is cited as a form of secondary obesity but there is no specification for surgery.
Possible limitations for Prader–Willi	Decisional capacity Adherence to post-operative treatment	N/A	N/A	N/A

ESPGHAN = European Society for Pediatric Gastroenterology Hepatology and Nutrition; ASMBS = American Society of Metabolic and Bariatric Surgery; IFSO = International Federation for the Surgery of Obesity and Metabolic Disorders; IFSO-EC = International Federation for the Surgery of Obesity—European Chapter; EASO = European Association for the Study of Obesity; SIEDP = Italian Society of Pediatric Endocrinology and Diabetology; SIP = Italian Society of Pediatrics.

## Data Availability

Not applicable.
